# Thrombosis of Abdominal Aorta and Bilateral Renal Arteries: Endovascular Treatment in Takayasu Disease

**DOI:** 10.7759/cureus.34409

**Published:** 2023-01-30

**Authors:** Ahmed Albaqshi, Mahdi Aljawad, Sara Alrasheed, Abeer Alshaia, Shaker Alshehri

**Affiliations:** 1 Radiology, National Guard Health, Riyadh, SAU

**Keywords:** case report, endovascular procedures, thrombosis, renal arteries, abdominal aorta, takayasu disease

## Abstract

Takayasu arteritis is an idiopathic vasculitis that typically involves the aorta and its major branches. It is more common in women and has the highest prevalence in Asia. Imaging studies are crucial for establishing the diagnosis and for determining the extent of the disease. We present the case of a 47-year-old man who presented with a complaint of anuria and generalized weakness for the last three days. He reported a history of generalized abdominal pain for the last two weeks. His vital signs were within normal limits, but the systolic blood pressure in the lower limb was lower by 60 mmHg compared with that of the upper limb. Notably, the pulses were very faint on palpation. Laboratory investigations revealed deranged renal function parameters. Ultrasound examination showed increased renal parenchymal echogenicity bilaterally with elevated peak systolic velocity of the main renal artery on spectral Doppler. Further investigation by computed tomography demonstrated near-complete thrombosis of the abdominal aorta distal to the origin of the celiac artery and extending to the common iliac arteries with the involvement of bilateral renal arteries. Immunological investigations, including antinuclear antibody (ANA), double-stranded deoxyribonucleic acid (dsDNA), cyclic antineutrophil cytoplasmic antibody (c-ANCA), and perinuclear antineutrophil cytoplasmic antibody (p-ANCA), revealed negative results. However, the positron emission tomography showed markedly diffuse and circumferential increased uptake in the walls of the aorta, subclavian arteries, and femoral arteries. The patient underwent successful endovascular treatment with catheter-directed thrombolysis. High clinical suspicion is required to identify renal artery thrombosis since the clinical symptoms are non-specific. Early diagnosis is crucial to allow for prompt therapeutic interventions.

## Introduction

Takayasu arteritis is a large-vessel vasculitis, with unclear etiology, that typically involves the aorta and its major branches. It has a worldwide distribution with the highest prevalence in Asia [1]. The age of onset is usually below the age of 40 years and the majority of patients are women. The diagnosis of Takayasu disease is made based on the suggestive clinical and imaging features. The clinical features include constitutional symptoms, hypertension, and diminished pulses. Imaging studies are crucial for establishing the diagnosis and for determining the extent of the disease. The imaging studies may include computed tomography angiography or magnetic resonance angiography to evaluate for arterial stenosis. Further, positron emission tomography is increasingly being utilized to evaluate large-vessel involvement [2]. The cornerstone therapy of Takayasu disease is glucocorticoid therapy [3]. Endovascular interventions may be needed in the management of critical ischemia due to irreversible arterial stenosis [4]. Here, we present the case of a middle-aged man presenting with anuria who was found to have Takayasu disease and managed with endovascular intervention. This case was previously presented as an abstract at the 2022 Pan Arab Interventional Radiology Society (PAIRS) Annual Congress on May 11-14, 2022.

## Case presentation

We present the case of a 47-year-old man who presented to the emergency department with a complaint of not producing any amount of urine and generalized weakness for the last three days. He reported no history of other urinary symptoms. Specifically, there was no history of dysuria, hematuria, or flank pain. He reported a history of generalized abdominal pain for the last two weeks, which was attributed to constipation and he was prescribed symptomatic treatment with laxatives. Further, the patient gave a history of bilateral calf pain after walking for a distance of 30 meters. There was no history of fever, anorexia, or weight loss. The past medical history of the patient was remarkable for hypertension and degenerative disc disease of the lumbar spine. Further, he had a history of an unprovoked pulmonary embolism that was treated with warfarin for five years. He did not have any previous surgeries. His current medications include aspirin, valsartan, metoprolol, and atorvastatin. The social and family history was non-contributory.

On examination, the patient was alert and conscious and did not appear in pain or distress. His vital signs were within the normal limits, including a body temperature of 36.3°C, pulse rate of 79 bpm, blood pressure of 127/80 mmHg, and respiratory rate of 20 bpm. There was a discrepancy in the blood pressure between the upper and lower limbs with the systolic blood pressure in the lower limb being lower by 60 mmHg compared with that of the upper limb. A cardiac examination revealed normal heart sounds with no added sounds or murmurs. The jugular venous pressure was not elevated and there was no carotid bruit noted. Examination of the peripheral pulses revealed an absent left radial artery pulse with weak lower limb pulses. The peripheral pulses were generally of low volume and difficult to palpate. Abdominal examination revealed mild tenderness in the right flank. However, no clinical signs of peritonitis were evident. The musculoskeletal system examination was normal. Furthermore, neurological examination revealed normal tone, muscle power, coordination, and reflexes. There was no sensory deficit at any level.

Initial hematological investigations revealed normal hemoglobin level (13.6 g/dL), leukocytosis (7100/μL), and normal platelets (302,000/μL). Renal function profile revealed elevated creatinine (500 μmol/L) and blood urea nitrogen (8 mmol/L), and normal electrolytes. The erythrocyte sedimentation rate was elevated (149 mm/hour).

**Figure 1:**
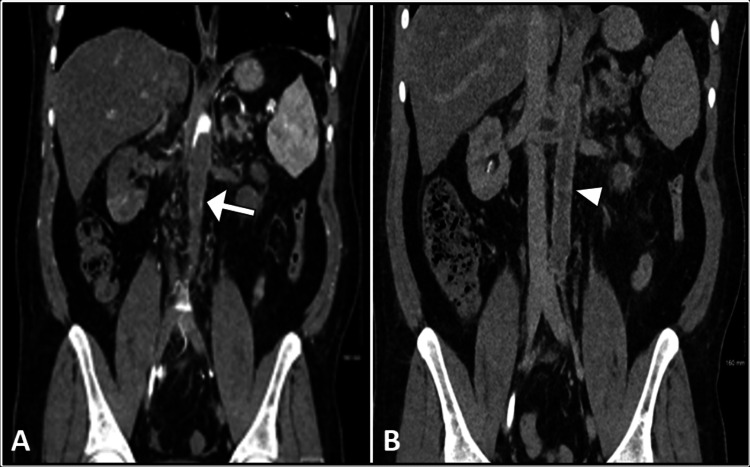
Coronal CT images show filling defects in the infrarenal abdominal aorta (arrow) in the portal venous phase (A). Enhancement of the abdominal aorta (arrowhead) is noted in the delayed phase (B). CT: computed tomography

He underwent a non-contrast computed tomography scan of the urinary tract which showed no evidence of obstructive uropathy or any density within the urinary tract to suggest stones. However, the scan showed perinephric fat stranding that could be related to recently passed stones. On the following day, the patient continued to have deterioration in renal function as the creatinine level almost doubled (900 μmol/L). Subsequently, an ultrasound examination of the kidneys showed increased renal parenchymal echogenicity bilaterally with elevated peak systolic velocity of the main renal artery with a pulsus parvus et tardus waveform on spectral Doppler. Such findings were concerning for renal artery stenosis. Further investigation by contrast-enhanced computed tomography scan demonstrated near-complete thrombosis of the abdominal aorta distal to the origin of the celiac artery and extending to the common iliac arteries with the involvement of bilateral renal arteries (Figure 1). Also, multiple wedge-shaped hypodense regions in the spleen and kidneys were noted as suggestive of ischemic infarcts.

The aforementioned clinical and imaging findings raised the possibility of vasculitis, which warranted further investigation and workup. The autoimmune profile, including antinuclear antibody, anti-double-stranded deoxyribonucleic acid (anti-dsDNA), anticardiolipin antibody, antiphospholipid antibody, anti-glomerular basement membrane (anti-GBM) antibody, cyclic antineutrophil cytoplasmic antibody (c-ANCA), and perinuclear antineutrophil cytoplasmic antibody (p-ANCA), yielded negative results. Further, gene testing for mutations in Janus kinase 2 (JAK2), prothrombin, and factor V Leiden genes was negative. Then, the patient was referred to undergo positron emission tomography with computed tomography. It showed markedly diffuse and circumferential increased uptake in the walls of the aorta, subclavian arteries, and femoral arteries.

**Figure 2:**
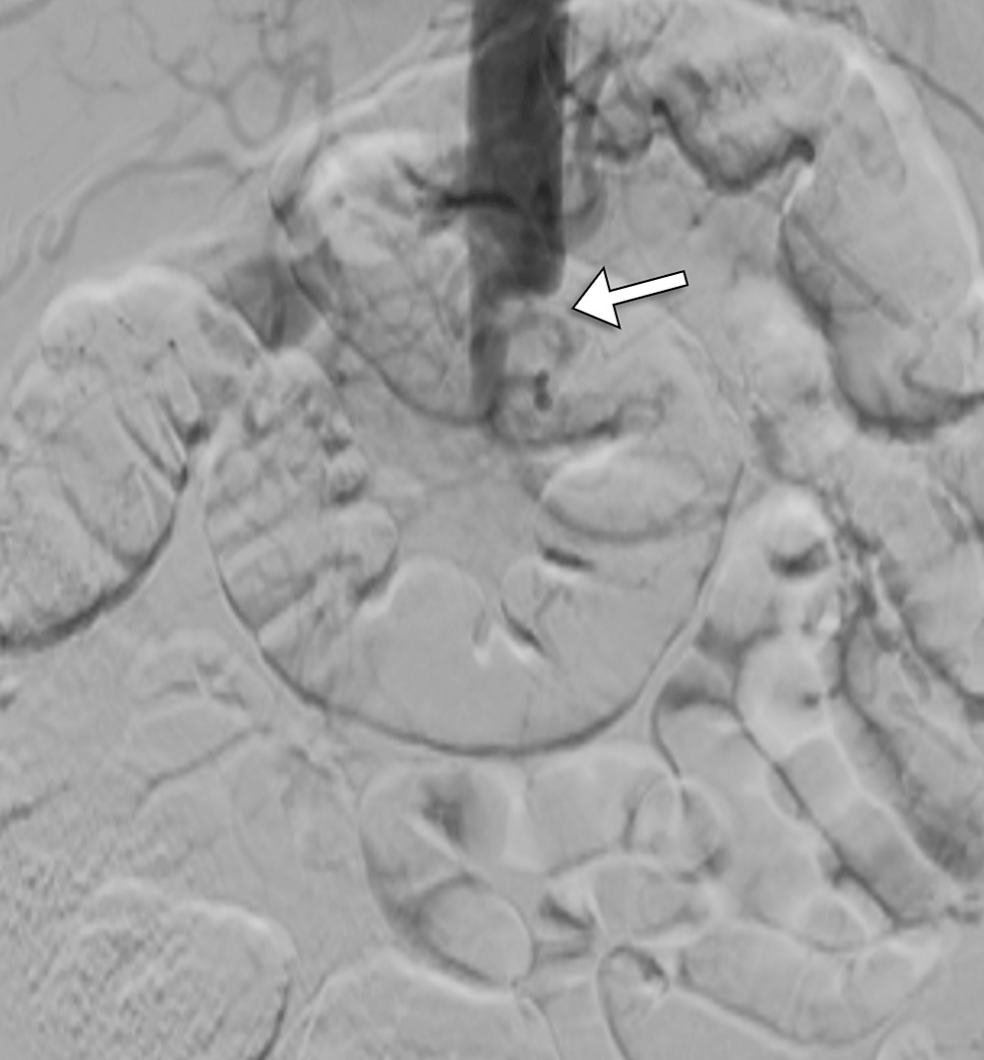
Selected DSA image shows an abrupt cut-off (arrow) in the infrarenal abdominal aorta representing thrombosis. DSA: digital subtraction angiography

**Figure 3:**
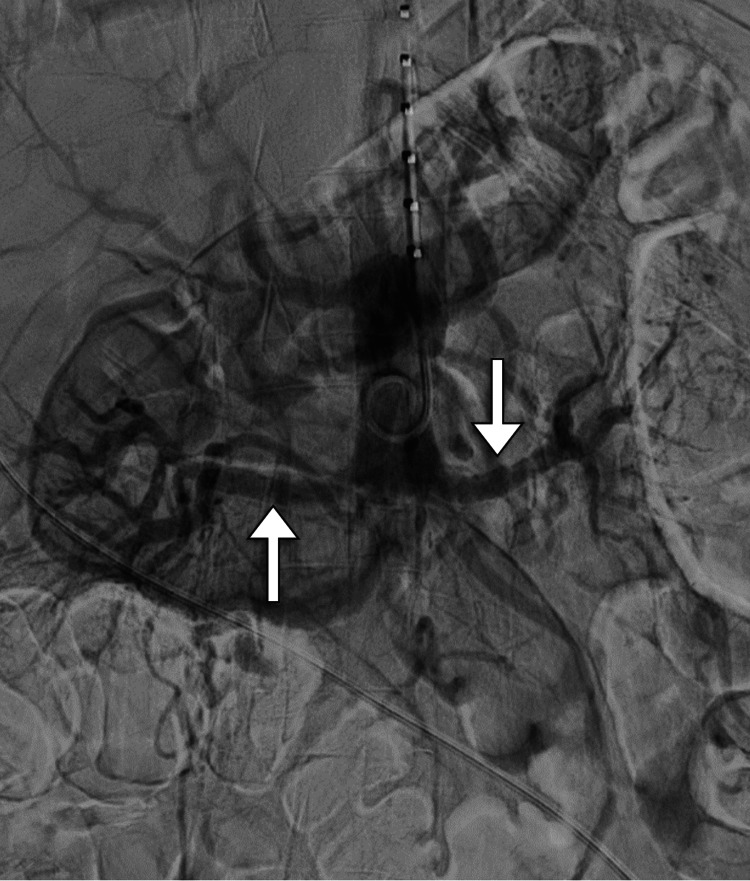
Selected DSA image shows complete patency of renal arteries bilaterally (arrows). DSA: digital subtraction angiography

**Figure 4:**
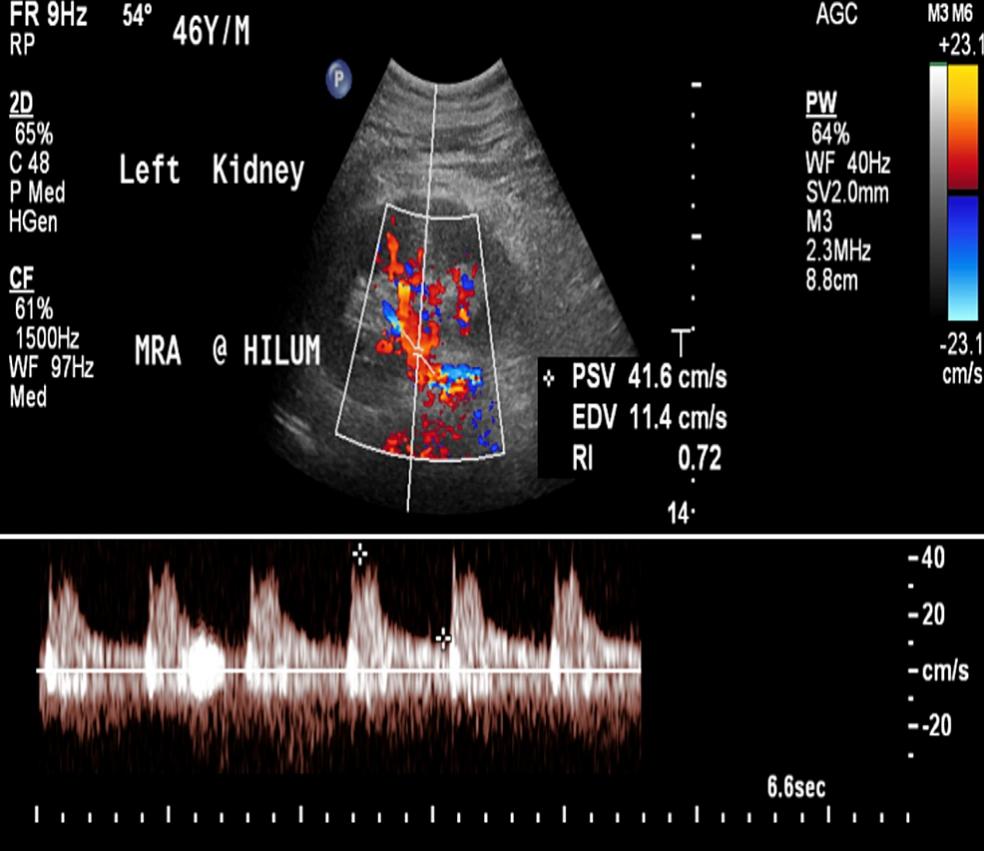
Ultrasound image with power Doppler showing normal flow in the renal artery.

The interventional radiology team advised catheter-directed thrombolysis of the renal arteries to salvage the kidneys. Under sterile conditions and with imaging guidance, access through the left brachial artery was obtained and a 5-French vascular sheath was inserted. A pigtail catheter was positioned at the abdominal aorta and the aortogram was obtained. The aortogram showed a complete aortoiliac occlusion distal to the celiac artery origin with no opacification of renal arteries (Figure 2). Then, the right renal artery was successfully recanalized and a 6 mg tissue plasminogen activator (t-PA) was administered as a bolus. Catheter-directed thrombolysis was placed in the right renal artery. After obtaining left radial artery access and insertion of the 5-French vascular sheath, the left renal artery was successfully recanalized and 6 mg of t-PA was administered as a bolus. The catheter-directed thrombolysis was placed in the left renal artery with its proximal end at the level of the suprarenal artery. Both catheters and vascular sheaths were fixed. The catheter-directed thrombolysis began with t-PA infusion through the catheter and heparin infusion through the sheaths. Follow-up angiography demonstrated complete patency of bilateral renal arteries with the resumption of flow into renal parenchyma (Figure 3). In addition, ultrasound examination with Doppler study of kidneys yielded normal results with no evidence of renal vascular occlusion (Figure 4).

Following the catheter-directed thrombolysis, the patient produced good urine output (3 liters/day). Anticoagulation with intravenous heparin, then warfarin 3 mg/day with an international normalized ratio target of 2-3 indefinitely. Prednisolone was administered with a dose of 25 mg/day and tapered down weekly by 5 mg to reach a dose of 5 mg/day. Methotrexate was initiated as an induction therapy as well, with a dose of 10 mg/week. At the follow-up visit after four months, the patient had normal creatinine levels.

## Discussion

We presented the case of Takayasu disease involving the renal arteries which were successfully managed by catheter-directed thrombolysis. The common differential diagnoses for a patient with renal artery thrombosis include cardiac emboli due to atrial fibrillation or endocarditis, vasculitis, sickle cell disease, fibromuscular dysplasia, and antiphospholipid syndrome [5]. However, these conditions were ruled out after appropriate investigations.

Vascular intervention may be necessary for the treatment of stenosed arteries leading to organ ischemia or hypertension and for the management of aneurysmal disease. The management of renal artery thrombosis and acute renal infarction remains controversial [5]. The open surgical procedure was found to be less effective than conservative management. The treatment with oral anticoagulant and systemic thrombolysis is a feasible option. Further, as in the present case, catheter-directed thrombolysis is a safe and effective therapeutic option. However, the use of catheter-directed thrombolysis has been described in limited case reports and series [6]. While it can successfully restore renal blood flow, the long-term outcomes are difficult to evaluate. Notably, the measurement of creatinine level is not a reliable reflector of the renal function of the affected kidney [7]. However, in the present case, both kidneys were affected and the creatinine clearance normalized at the follow-up visit after the discharge.

Acute occlusion of the renal artery is an uncommon clinical entity with significant morbidity and mortality [6]. The majority of cases present with hypertension, fever, nausea, hematuria, and acute renal failure. As in the present case, the clinical diagnosis is often challenging as the clinical manifestations are non-specific.

## Conclusions

The case highlighted the successful use of catheter-directed thrombolysis in the management of bilateral renal artery thrombosis in a patient with Takayasu disease followed by subsequent oral anticoagulation therapy. High clinical suspicion is required to identify renal artery thrombosis since the clinical symptoms are non-specific. Early diagnosis is crucial to allow for prompt therapeutic interventions.
